# Comparison of fundus fluorescein angiography, optical coherence tomography and optical coherence tomography angiography features of macular changes in Eales disease: a case series

**DOI:** 10.1186/s12348-020-00220-4

**Published:** 2020-12-14

**Authors:** Ketaki Rajurkar, Meenakshi Thakar, Priyadarshi Gupta, Anju Rastogi

**Affiliations:** Guru Nanak Eye Centre, Near Zakir Hussain College, Maharaja Ranjeet Singh Marg, 64 Khamba, New Delhi, Delhi, 110002 India

**Keywords:** Eales disease, Slit lamp bio microscopy, Optical coherence tomography, Optical coherence tomography angiography, Deep capillary plexus

## Abstract

**Purpose:**

To study the macular features in Eales disease patients observed with fundus fluorescein angiography (FA), optical coherence tomography (OCT) and optical coherence tomography angiography (OCTA).

**Methods:**

A cross-sectional study was done on treatment naïve 31 eyes (23 patients) with Eales disease. Baseline parameters such as Best-corrected visual acuity (BCVA), slit-lamp bio microscopy (SLB), indirect ophthalmoscopy, FA, spectral-domain OCT {quantitative (central macular thickness [CMT]) and qualitative analysis on SD-OCT} and OCTA were performed. Any media opacity precluding the above investigations was excluded.

**Results:**

Macular findings comprised of- epiretinal membrane, macular exudation, full thickness macular hole, sub internal limiting membrane bleed, cystoid macular oedema, neurosensory detachment and retinal thickening. Sixteen (51.6%) of our patients had macular changes as seen on all modalities together. SLB and indirect ophthalmoscopy missed macular findings in 50% patients and FA in 18.8% patients. OCT and OCTA diagnosed all macular findings. On comparison of mean BCVA in patients with macular involvement on FA, OCT and OCTA, compared to those without macular involvement, patients with macular involvement had lower BCVA (p 0.000, 0.01 and 0.001 respectively). Thus, FA missed many patients who had significant macular involvement and hence less vision.

**Conclusion:**

Eales disease though described in literature as classically being peripheral retina disease process, also has macular involvement. OCT and OCTA are useful guides to evaluation of macular involvement in these patients. The latter seems to be superior to FA in detecting macular abnormalities in this ailment. OCTA is non-invasive and shows deep capillary plexus changes which are not shown by any other modality.

## Introduction

Eales disease was first described by the British ophthalmologist Henry Eales in 1880. Eales disease is an idiopathic, occlusive perivasculitis affecting the peripheral retina, leading to retinal non-perfusion, new vessel formation, and recurrent vitreous hemorrhages. There is a vicious cycle of retinal inflammation, hypoxia and neovascularization [1, 2]. It mostly affects healthy young men, aged 20–40 years and is a diagnosis of exclusion. It has been reported from the United Kingdom, the United States, and Canada in the latter half of 19th and early twentieth century. However, it is more commonly reported from the Indian subcontinent. The reported incidence in India is 1 in 135 ophthalmic patients in a referral ophthalmic center and 1 in 200 to 250 in a general eye hospital [3]. Since it has been classically described as peripheral vasculitis, macular changes have been reported infrequently in literature. Macular findings have been reported more in recent literature since the advent of better imaging techniques. Some studies have reported that macular changes occur in approximately 18% of eyes with Eales disease, the most common of which is macular edema. Intravitreal anti vascular endothelial growth factor (VEGF) has been used for treatment of macular oedema. Vitrectomy with epiretinal membrane (ERM) peel has been described for ERM in Eales disease [2, 4–8].

Optical Coherence Tomography Angiography (OCTA) is a novel modality used to study the retinochoroidal vasculature in detail. It is non-invasive and does not require application of intravenous dye. It uses motion contrast imaging to high-resolution volumetric blood flow information generating angiographic images within seconds. OCTA compares the decorrelation signal (differences in the backscattered OCT signal intensity or amplitude) between sequential Optical coherence tomography (OCT) b-scans taken at precisely the same cross-section in order to construct a map of blood flow. Pooling or staining does not occur in OCTA, since there is no dye leaking from pathological microvasculature. This allows better visualization of the whole microvasculature including the foveal avascular zone (FAZ). It has also been reported that deep capillary plexus changes are demonstrated on OCTA but FA is not able to demonstrate these changes [9, 10].

India has a high incidence of Eales disease as mentioned above. Since there is infrequent reporting of macular findings in these patients, we conducted this study. There is also scarcity of data in literature about the OCT and OCTA findings of these patients. Our present study throws light on importance of these imaging techniques in Eales disease [8].

## Methods

A prospective cross-sectional study was done on treatment naïve 31 eyes of 23 patients with Eales disease presenting to retina clinic of tertiary care hospital. Informed consent was obtained from all the patients. Permission for conducting the study was obtained from Institutional review board.

Demographic profile of the patient such as age, gender and eye involved was noted. Baseline parameters such as Best-corrected visual acuity (BCVA), slit-lamp bio microscopy (SLB), intraocular pressure and indirect ophthalmoscopy (IO) were done.

Peripheral retinal periphlebitis of small and large caliber vessels with peripheral capillary nonperfusion, and/ or neovascularization elsewhere/of the disc with secondary fibrovascular proliferation on FA was described as peripheral vasculitis. Other findings such as vascular sheathing, perivascular exudates and superficial retinal hemorrhages were noted. All other causes of retinal vasculitis were excluded.

Systemic diseases causing similar presentation such as diabetic retinopathy, vascular occlusion, raised serum homocysteine levels, Sarcoidosis, Syphilis, blood dyscrasias, Coats disease, Familial exudative vitreoretinopathy (FEVR), Acquired immunodeficiency syndrome, Behcet’s disease and Sickle-cell disease were excluded. All relevant investigations such as- Complete haemogram with erythrocyte sedimentation rate, peripheral smear, Venereal disease research laboratory test, serum angiotensin converting enzyme levels, Human Immunodeficiency Virus, Hepatitis C Virus, Hepatitis B surface antigen, blood sugar level, serum calcium level, Mantoux test and X-ray chest were done.

Fundus fluorescein angiography (FA) (VISUPAC and FF450plus, Carl Zeiss Meditec, Inc., Dublin, California, USA.) was done. All the relevant findings such as peripheral retinal periphlebitis, peripheral capillary non perfusion, foveal avascular zone abnormalities, and neovascularization elsewhere and of the disc, collateral formation was noted. Macular Ischemia on fluorescein angiography was defined as enlargement of the FAZ and/or capillary dropout encroaching on the FAZ.

Spectral domain- optical coherence tomography (RS-3000 Nidek Gamagori, Japan) was used to study the macular changes. ETDRS grid includes 9 quadrants within 6 mm of foveal center formed by dividing this area by 1 mm and 3 mm distance concentric circles. This was used for central macular thickness (CMT) quantitative measurement. Qualitative changes including retinal thickening, cystoid macular oedema, hard exudates, ERM, neurosensory detachment and intra or sub retinal bleed were noted. The data was differentiated into normal or abnormal based on comparison with age matched data of normal patients.

Optical coherence tomography angiography (RS-3000 Advance, Nidek Gamagori, Japan) was performed to study superficial retina, deep retina, retinal pigment epithelium (RPE) level and choroid. Superficial and deep retinal plexus morphology was studied in detail. Quantitative measurement of superficial retinal capillary plexus (SCP) and deep capillary plexus (DCP) area on OCTA was done.

All the findings on FA, OCT and OCTA were evaluated and reported by two independent observers (KR and MT). The findings were noted to be reproducible, especially OCT and OCTA since they could be repeated every visit.

Macular involvement definition: Macular involvement was defined on FA as macular leakage, enlarged FAZ, capillary dropout at FAZ or epiretinal membrane. Macular involvement on OCT was defined in the form of changes including retinal thickening, cystoid macular oedema, hard exudates, ERM, full thickness macular hole (FTMH), neurosensory detachment and intra or sub retinal bleed at macula.

Patients were classified according to Eales’ disease classification system [7]. (Eur J Ophthalmol 2004; 14: 236–9):

- Stage 1a: Periphlebitis of small caliber vessels with superficial retinal hemorrhages
- Stage 1b: Periphlebitis of large caliber vessels with superficial retinal hemorrhages
- Stage 2a: Peripheral capillary non-perfusion
- Stage 2b: Neovascularization elsewhere/Neovascularization of the disc
- Stage 3a: Fibrovascular proliferation
- Stage 3b: Vitreous hemorrhage

Exclusion criteria:

1. Any media opacity precluding the above investigations, especially OCTA which requires good media clarity, was excluded.
2. Stage 4 disease patients according to Eales’ disease classification system [7] were excluded from the study in view of poor visualization of retinal layers (in most of the presenting cases) which is essential for OCT and OCTA.

Outcome measures included:

Primary:

1. Comparison of mean Logmar BCVA among patients with macular changes in FA, OCT and OCTA.
2. Calculation of SCP and DCP area and its correlation with macular changes on OCT.

Secondary:

1. Comparison of qualitative changes on FA, OCT and OCTA in macula.
2. Comparison of CMT on OCT in patients with macular involvement and those without macular involvement.
3. Qualitative changes on OCT in patients with macular involvement and comparison with those without macular involvement.
4. Qualitative changes on OCTA in patients with macular involvement and comparison with those without macular involvement.

Statistical analysis was done using SPSS 16 (SPSS Inc., Chicago, Illinois, USA). The data was presented as mean ± standard deviation. Mann Whitney U test was used for analysis of quantitative variables such as BCVA, CMT and superficial and deep capillary plexus area between the groups. *P* < 0.05 was considered statistically significant.

## Results

Thirty-one eyes of 23 consecutive patients with Eales disease were included.

### Demography

Baseline characteristics of study population are given in Table 1. The baseline Logmar BCVA ranged from 0.0 to 1.8 (Mean: 0.5 ± 0.43).

**Table 1:** Baseline characteristics of patients

**Total eyes**	31
**Mean age**	24.87 ± 5.7 years (17 to 36 years)
**Gender**	
Male	27 male (87.1%)
Female	4 female
**Bilateral involvement**	8 patients
**Known history of Tuberculosis**	3 patients (13%)
**Family history of Tuberculosis**	7 patients (30.4%)
**BCVA**	0.0 to 1.8 (Mean: 0.5 ± 0.43)

Seven patients (35%) had a family history of tuberculosis in close family members living with them. Three patients were known cases of tuberculosis on anti-tubercular treatment.

Sixteen (51.6%) patients had macular changes as seen on all modalities together. SLB and IO missed the finding in 8 patients (50%) out of total patients with macular involvement. FA missed macular findings in 3 (18.8%) patients. OCT and OCTA demonstrated the findings in 100% patients (Table 2).

**Table 2:** Number of patients with macular changes

Total patients with macular changes	16 patients (51.6%)
Patients with macular changes on SLB and indirect ophthalmoscopy	8 patients (50%)
Patients with macular changes on FA	13 patients (81.25%)
Patients with macular changes on OCT and OCTA	16 patients (100%)

Other than ischemic changes at macula, FA also demonstrated changes such as leakage at macula, window defect in FTMH, peripheral periphlebitis, peripheral capillary non perfusion and neovascularization elsewhere and of the disc.

**Fig. 1:**
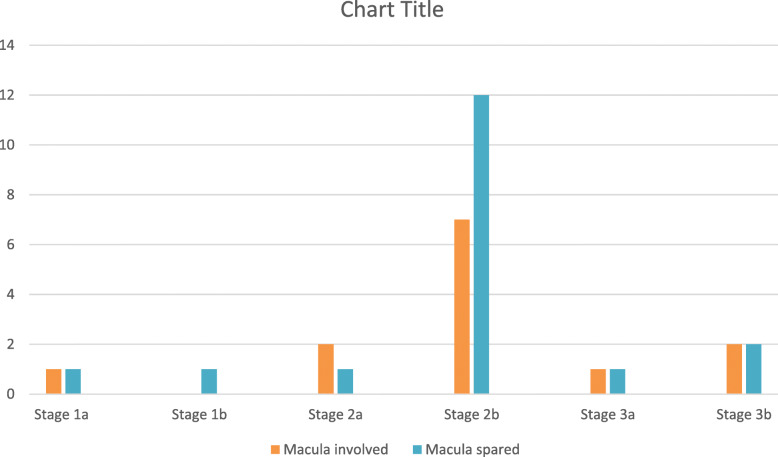
Classification of cases according to Eales’ disease classification system. (Eur J Ophthalmol 2004; 14: 236–9)

Classification of cases according to Eales’ disease classification system [7] (Eur J Ophthalmol 2004; 14: 236–9): The classification of cases is given in Table 3. Stage 2b had the highest number of cases and also maximum cases with macular involvement. Due to the small number of cases in each group, intergroup comparison was not done (Fig. 1).

**Table 3:** Classification of cases according to Eales’ disease classification system. (Eur J Ophthalmol 2004; 14: 236–9)

Stages	Number of cases
Stage 1a	2 patients (1 patient with macular involvement)
Stage 1b	1 patients (No macular involvement)
Stage 2a	3 patients (2 patients with macular involvement)
Stage 2b	19 patients (7 patients with macular involvement)
Stage 3a	2 patients (1 patients with macular involvement)
Stage 3b	4 patients (2 patients with macular involvement)

### Primary outcome

The comparison of eyes with macular involvement on FA, OCT and OCTA in terms of mean Logmar BCVA was done (Table 4).

**Table 4:** Comparison of mean BCVA among patients with or without macular involvement on FA, OCT and OCTA

IMAGING MODALITY	Macular involvement	Number of patients	BCVA	*P* value
FA	Macula involved	10 patients	0.87 ± 0.44 (0.48 to 1.8)	0.000
	Macula non-involved	21 patients	0.33 ± 0.31 (0.0 to 1.18)	
OCT	Macula involved	13 patients	0.7 ± 0.48 (0.0 to 1.8)	0.01
	Macula non-involved	18 patients	0.36 ± 0.33 (0.0 to 1.8)	
OCTA	Macula involved	12 patients	0.8 ± 0.46 (0.3 to 1.8)	0.001
	Macula non-involved	19 patients	0.32 ± 0.3 (0.0 to 1.18)	

We compared the BCVA between the eyes with macular involvement on FA versus eyes with no involvement of macula on FA. The *p* value was statistically significant (*p* = 0.000). Thus, FA was able to accurately predict macular changes and corresponding visual acuity reduction. We also compared the mean BCVA between the eyes with macular involvement on OCT versus eyes with no involvement of macula on OCT. The p value was statistically significant (*p* = 0.01).

On comparison of eyes with macular involvement on OCTA versus eyes with no involvement of macula on OCTA with mean BCVA, eyes with involvement had significantly lower BCVA than those with normal macula (p 0.001).

### OCTA findings and SCP-DCP correlation with macular changes on OCT

**Fig. 2:**
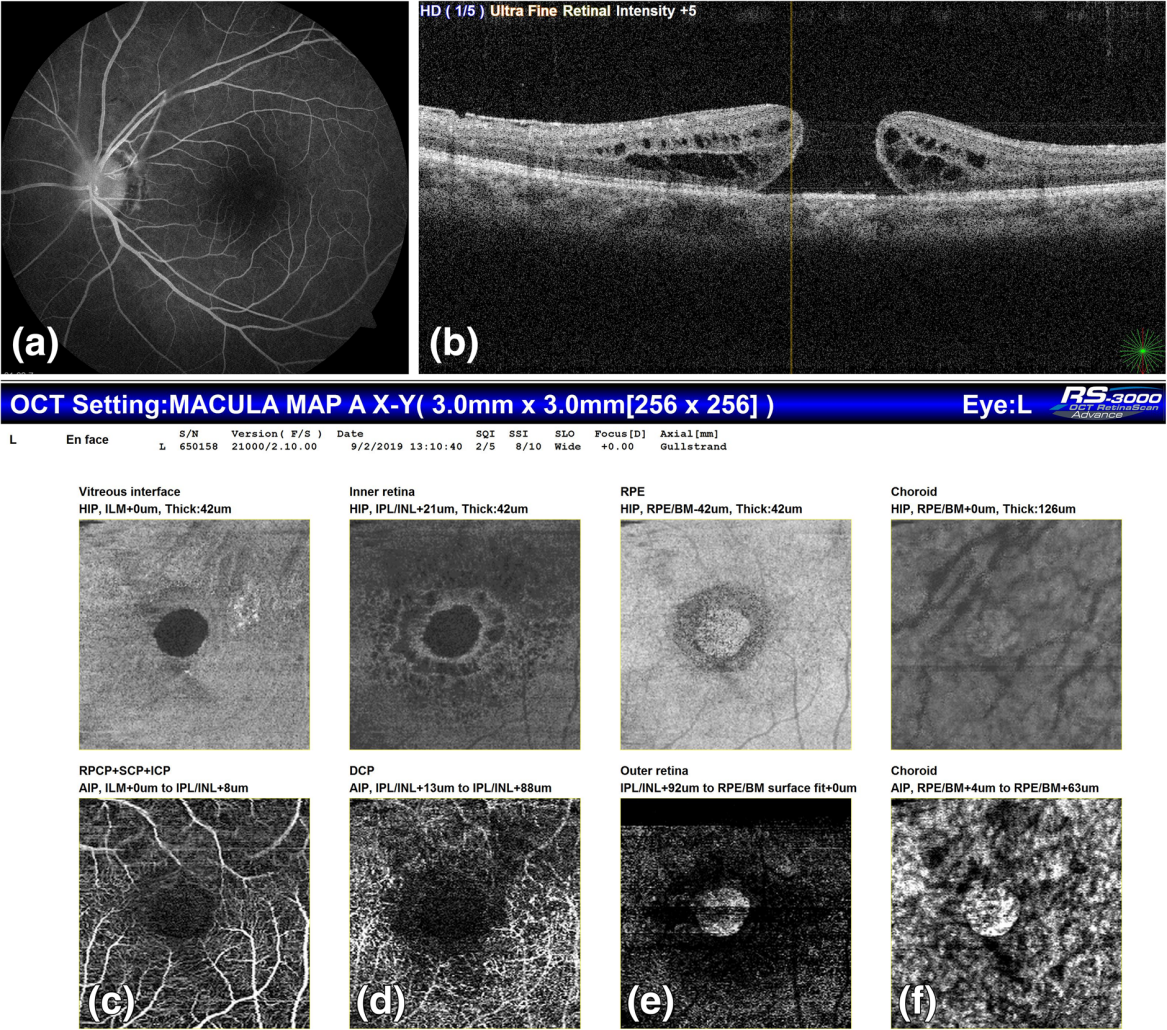
36 years old male with vision in left eye counting finger 3 m; (a) FA: window defect at macula due to FTMH (b) OCT: shows FTMH with cuff of fluid (c) OCTA at SCP (d) OCTA at DCP (e) OCTA at outer retina (f) OCTA at choroid level shows FTMH with back shadowing

**Fig. 3:**
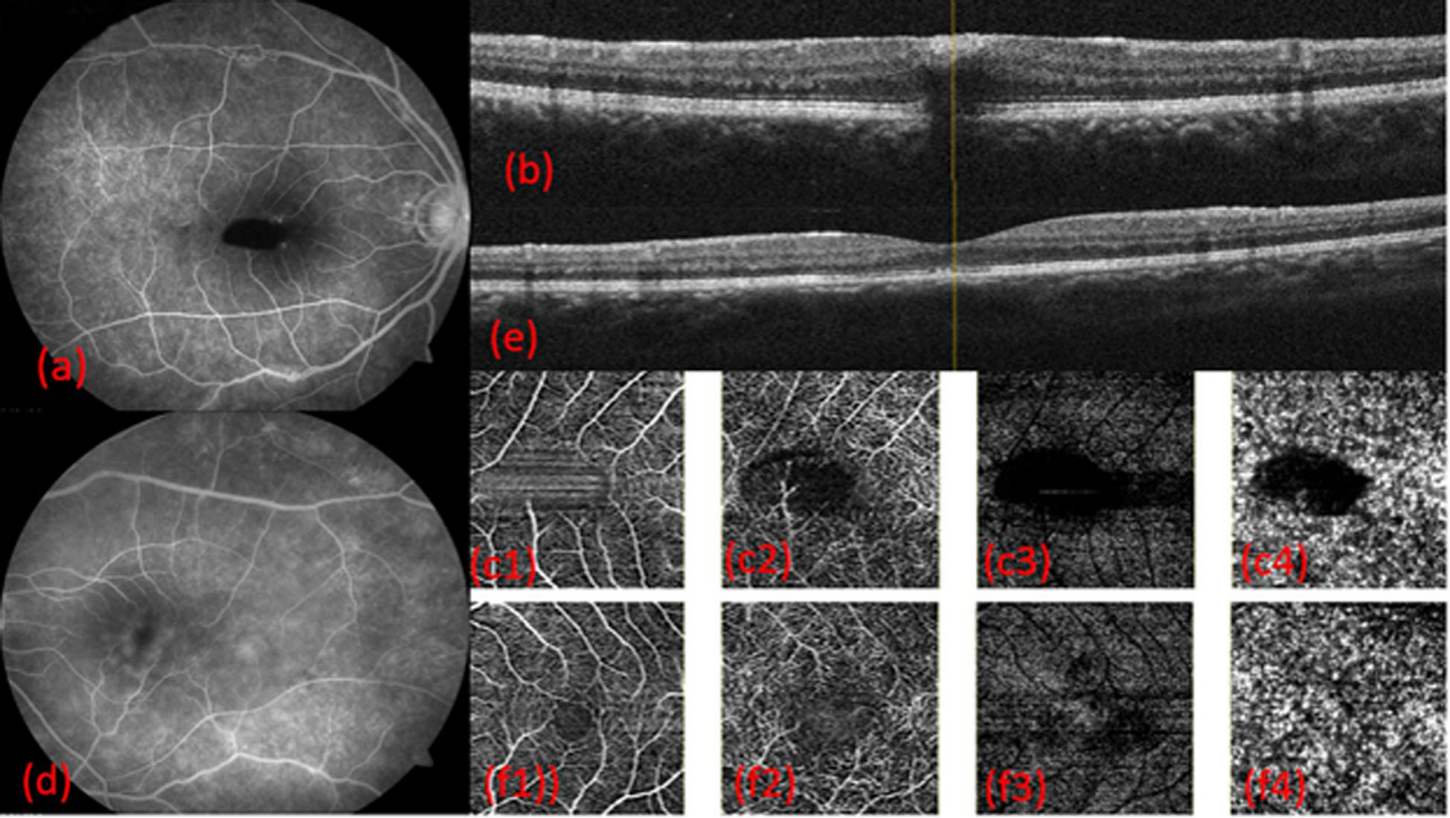
17 years male vision right eye counting finger 1 m and left eye 6/24; (a) FA: Right eye with blocked fluorescence (b) OCT: shows sub-ILM bleed (c1–4) OCTA at SCP, DCP, outer retina and choroid shows back-shadowing due to bleed (d) FA: Left eye with leakage at macula (e) OCT shows outer retina mild irregularity (f1–4) OCTA at SCP,DCP, outer retina and choroid level suggestive of DCP enlargement

It was noted in all the patients that only the retinal layers were involved on OCTA with sparing of RPE and choroid. In patients with FTMH and sub-ILM bleed, back shadowing was noted (Figs. 2 and 3). Average superficial and deep capillary plexus areas are given in Table 5.

**Table 5:** SCP-DCP area

RETINAL PLEXUS ON OCTA	AVERAGE AREA OF PLEXUS
SCP	0.39 ± 0.13 (Range 0.18 to 0.79) μ
SCP area in eyes with macular involvement	0.45 ± 0.14 (Range 0.28 to 0.79) μ
DCP	0.69 ± 0.23 (Range 0.31 to 1.49) μ
DCP area in eyes with macular involvement	0.88 ± 0.24 (Range 0.65 to 1.49) μ.

*SCP* Superficial capillary plexus; *DCP* Deep capillary plexus area

On performing statistical analysis, the correlation of superficial plexus area with the macular involvement was not statistically significant (p 0.12). However, the deep plexus area and macular involvement was significantly positively correlated (p 0.00).

### Secondary outcomes

Table 6. Data of patients with macular involvement on FA, OCT and OCTA. Comparison of the findings on these three modalities is given for all the individual patients.

**Table 6:** Data of patients with macular involvement on FA, OCT and OCTA

***No.***	***Age (years)***	***Gender***	***BCVA***	***Changes in macula on FA***	***CMT***	***OCT***	***OCTA***
1.	17	M	0.3	Sub ILM bleed-blocked fluorescence	368	Sub-ILM bleed	Back shadowing of bleed
2.	20	M	1	WNL	308	WNL	Enlarged deep capillary plexus
3.	22	M	0.48	Ischemic macula	260	Few cystoid spaces	Enlarged deep capillary plexus
4.	23	M	0.6	Ischemic macula	307	Diffuse retinal thickening	Distorted plexus
5.	23	M	0.78	Ischemic macula	282	Diffuse retinal thickening	Distorted plexus
6.	22	M	0.6	Distorted macular vessels, ERM	226	ERM blunted foveal contour, retinal thinning	Distorted plexus
7.	24	M	0.48	Ischemic macula	394	CME and NSD	Enlarged deep capillary plexus
8.	21	M	0.3	WNL	325	Diffuse retinal thickening	Enlarged deep capillary plexus
9.	18	F	1.3	Ischemic macula	303	Diffuse oedema in inferotemporal quadrant, hard exudates	Enlarged deep capillary plexus
10.	36	M	1.3	Window defect	FTMH	FTMH	Enlarged deep capillary plexus
11.	17	M	0.6	Leakage at macula	282	WNL	Enlarged deep capillary plexus
12.	35	M	0.3	WNL	283	WNL	Enlarged deep capillary plexus
13.	25	M	0.6	WNL	395	CME	WNL
14.	32	M	0	WNL	321	Diffuse retinal thickening	WNL
15.	25	M	0.3	WNL	310	Diffuse retinal thickening	WNL
16.	19	M	0.78	Ischemic macula, leakage at macula	191	Hard exudates at macula	WNL

*M* Male; *F* Female; *VN*: Vision; *CMT* Central macular thickness; *WNL* Within normal limit; *FTMH* Full thickness macular hole; *ERM* Epiretinal membrane; *ILM* Internal limiting membrane; *NSD* Neurosensory detachment

### Central macular thickness on OCT

Mean central macular thickness was 290.53 ± 48.28. Range was 191 to 395 μ. Mean CMT in eyes with macular involvement was 306.83 ± 62.10 μ. Mean CMT in eyes without macular involvement was 279.7 ± 34.18 μ. On comparison, the CMT in eyes with macular involvement was higher than eyes with no macular involvement but this finding was not statistically significant (p 0.09).

The commonest macular finding on OCT was diffuse retinal thickening. Other findings noted were cystoid macular oedema, sub-foveal detachment, epiretinal membrane, retinal thinning, hard exudates at macula, sub-internal limiting membrane bleed and FTMH.

### Comparison of foveal avascular zone on FFA and OCTA

We studied the foveal avascular zone in detail on FFA and OCTA. Any discrepancy between the two was noted. We observed that distorted FAZ on FA suggestive of ischemic maculopathy was seen in 5 patients. However, on OCTA, distorted superficial capillary plexus FAZ was seen in 3 patients and distorted deep capillary plexus FAZ was seen in 11 patients.

## Discussion

Our present study highlights the presence of macular changes as a common finding in Eales disease patients presenting to a tertiary care eye hospital. Macular involvement has been previously described in literature. In a study conducted in patients with Eales disease between 1989 and 1997, on SLB and FA, macular involvement was found in 28% of cases. The same authors, in a retrospective analysis, reported macular involvement seen in 32% cases in patients with Eales disease. Another study has reported macular involvement in 35.4% patients [11]. A recent study reported that 58.2% eyes of Eales disease patients had macular involvement as assessed with SD-OCT [8]. Thus, they noted macular changes to be a frequent finding. Macular oedema was the most common (35.4%) followed by ERM (11.4%) and macular thinning (11.4%). Other macular changes reported were hard exudates (6.3%), macular hemorrhages (5%), internal retina or internal limiting membrane folds (3.8%), pre macular hemorrhages (2.5%) and macular holes (1.2%). It has been reported that macula is usually not involved primarily in Eales disease despite extensive peripheral non-perfusion. But when it does get involved, it is termed central Eales disease. In this condition, all the classic mid-peripheral lesions appear at the posterior pole and cause decreased vision in the early stage of the disease, often due to cystoid macular oedema [12]. 51.6% of our patients had macular changes as seen on all modalities together. Many of our patients had classic Eales lesions at macula, thus suggestive of central Eales.

Macular changes in Eales disease have been increasingly reported in recent literature. We feel that one of the reasons for this is the advent of newer modalities like OCT and OCTA which give exact enface and cross-sectional images of the retina.

The age range reported in our study is in accordance with other studies which report the age range to be between 20 to 40 years [8, 12]. The male preponderance (87.1%) as seen in our study, has been reported previously [3].

The comparison of mean BCVA in patients with and without macular involvement diagnosed on FA, OCT and OCTA has scarcely been done before. We noted that the mean BCVA was less in patients with macular changes compared to those without macular changes (*p* < 0.05). This has been previously reported [8]. Though the *p* value was significant on all three modalities separately, however, FA missed some macular involvement patients which were picked up on OCT and OCTA. Thus, OCT and OCTA are very sensitive tools for analysis of macular changes in Eales disease and also predictors of visual acuity.

OCTA of Eales disease patients has scarcely been done [10]. In our study, SCP and DCP area was enlarged on OCTA in patients with macular involvement. The deep plexus area and macular involvement was highly significantly correlated (p 0.00). We concluded that the deep capillary plexus enlargement has more association with foveal involvement. The FAZ area calculation in Eales disease patients has never been reported before and since normative database of Indian eyes is not yet available, comparison of our data with normal could not be done. We also noted that RPE and choroid level OCTA scans were normal, thus suggestive of a retinal vascular pathology since it affected only the retinal layers.

It has been demonstrated that OCTA can be a better modality for macular changes, especially in the perifoveal area, than FA, since it distinguishes the different layers of retina and choroid, which cannot be done on FA. It also differentiates the superficial from deep plexus which is not possible on FA. Quantification of capillary plexuses can be done for follow up on OCTA and also visual prognostication. Pooling and leakage which can hamper macular analysis are not seen on OCTA thus making it a good modality for macular analysis. The adverse events related to dye injection are also averted on OCTA. However, periphery evaluation for neovascularization and capillary non-perfusion needs FA since it has a wider field of view [9, 10].

In our study, SLB and IO missed the finding in 8 patients (50%) out of the total patients with macular involvement. In another study [8], 28.3% were noted to have no macular changes based on SLB though they were noted on OCT. FA missed macular findings in 3 (18.8%) patients in our study. OCT and OCTA demonstrated the findings in 100% patients.

**Fig. 4:**
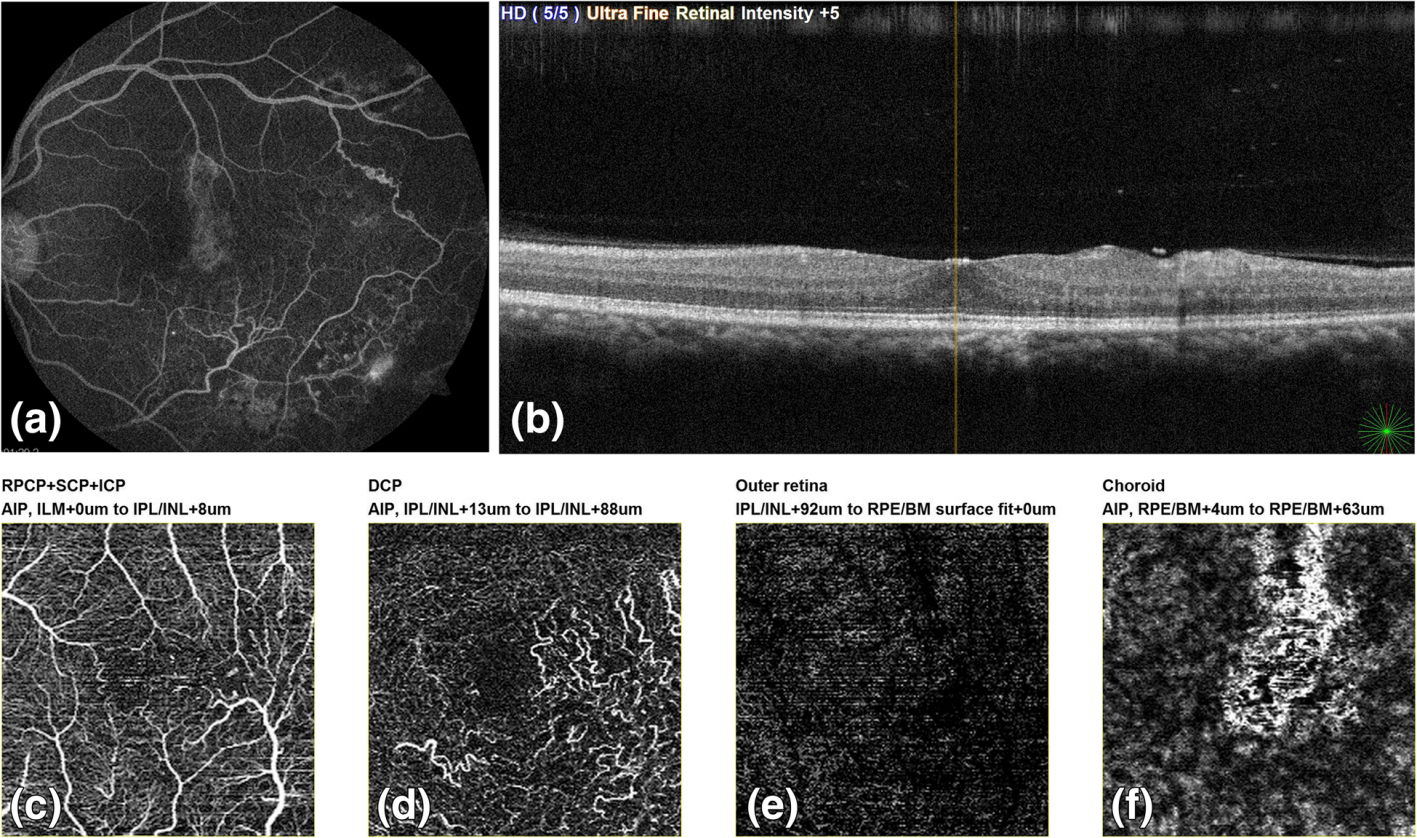
23 years male vision left eye 6/24; (a) FA: distortion at macula due to inferotemporal branch retinal vein occlusion with ERM (b) OCT: shows ERM (c) OCTA at SCP (d) OCTA at DCP (e) OCTA at outer retina (f) OCTA at choroid level shows irregularity of vessels at SCP and DCP

**Fig. 5:**
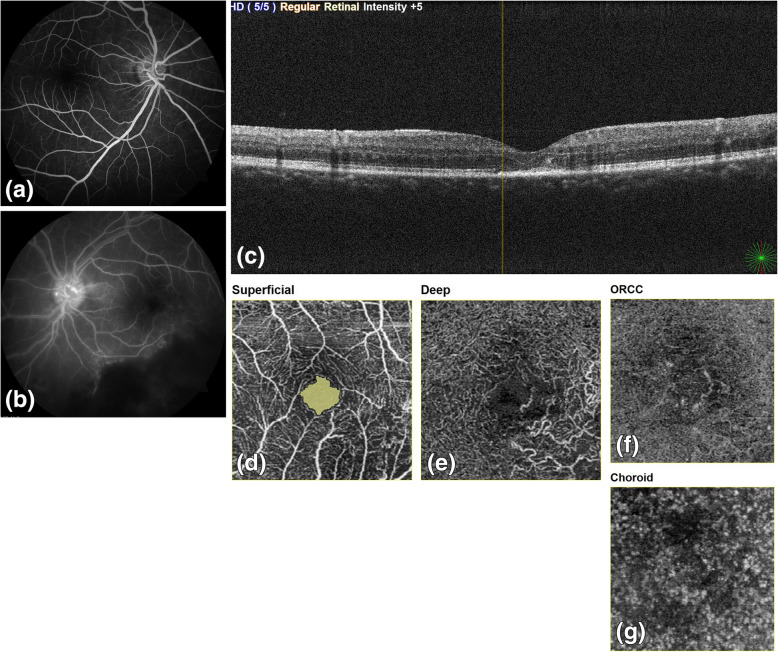
22 years male vision left eye 6/18; (a) FA right eye: WNL (b) FA left eye macula appears WNL, capillary non perfusion encroaching posterior pole; FAZ appears normal (c) OCT: Few cystoid spaces and mild distortion of outer retinal layers (d) OCTA at SCP shows manually marked area of SCP: WNL (e) OCTA at DCP which is enlarged (f) OCTA at outer retina (g) OCTA at choroid level

We noted that FA was able to identify vasculitis and leakage at macula accurately. However, macular changes in the form of capillary non perfusion, distorted FAZ especially at the deep capillary plexus were missed in 6 out of total 11 patients on FA. One patient with unexplained loss of vision with normal SLB and FA actually showed changes in DCP which explained the visual loss. DCP enlargement disproportionate to SCP enlargement was noted in 8 patients. These patients also had visual loss, thus, highlighting the importance of DCP involvement in Eales disease patients and its effect on their vision (Figs. 4 and 5). There have been previous reports highlighting the changes in DCP selectively in retinal venous occlusion corresponding with vision. It has also been reported that FA can identify SCP changes but not DCP changes separately since it is a two dimensional imaging modality [13–18].

We classified our cases according to Eales’ disease classification system [7] and observed that all the groups except stage 1b had macular involvement. Stage 2b had the highest number of macular involvement patients.

On comparison of CMT in eyes with macular involvement versus eyes without macular involvement, CMT of eyes with macular involvement was higher than eyes without it, but this finding was not statistically significant (p 0.09). This is in accordance with other studies [8]. The qualitative macular changes noted on OCT were diffuse retinal thickening, cystoid macular oedema, sub-foveal detachment, epiretinal membrane, retinal thinning, hard exudates at macula, sub-internal limiting membrane bleed and FTMH. Macular oedema as the commonest finding in Eales has been reported recently [7, 8, 11, 19]. Other changes have scarcely been reported, especially FTMH [8, 19]. Premacular haemorrhage and retinal thinning are also rare [8].

Our study included multimodal analysis of macular changes in Eales disease with SLB, IO, FA, OCT and OCTA which has never been done before. This, we feel, is the strength of our study. Our limitation is the small number of patients included. Eales disease being a rare entity and a diagnosis of exclusion, it is difficult to conduct large sample studies on these patients. Some patients in whom media is not clear, the above investigations cannot be conducted. This fact limits the number of study patients. A larger study may be able to establish the results demonstrated by our pilot study.

## Conclusion

Eales disease though described in literature as classically being a peripheral retina disease process, also has macular involvement especially in patients with central fundus findings and neovascularization. Macular oedema is the commonest macular abnormality in these patients. Macular involvement is associated with decreased visual acuity. Hence, it should be actively looked for in Eales disease. OCT and OCTA are useful guides to evaluation of macula and visual prognostication. OCTA, which is non-invasive, seems to be superior to FA in detecting macular abnormalities in this ailment. FAZ measurement for comparison can also be done with OCTA. It also shows DCP changes which are not shown by any other modality. In view of these advantages, OCT and OCTA should be routinely recommended for all Eales disease patients.

## Data Availability

The datasets used and/or analyzed during the current study are available from the corresponding author on reasonable request.

## Abbreviations

OCT: Optical coherence tomography
OCTA: Optical coherence tomography angiography
SLB: Slit lamp bio microscopy
IO: Indirect ophthalmoscopy
FA: Fluorescein angiography
FAZ: Foveal avascular zone
CMT: Central macular thickness
SCP: Superficial capillary plexus
DCP: Deep capillary plexus
LOGMAR BCVA: Logarithm of minimum angle of resolution best corrected visual acuity
ERM: Epiretinal membrane
RPE: Retinal pigment epithelium
